# Comparison of pain burden and psychological factors in Brazilian women living with HIV and chronic neuropathic or nociceptive pain: An exploratory study

**DOI:** 10.1371/journal.pone.0196718

**Published:** 2018-05-02

**Authors:** Andressa de Souza, Wolnei Caumo, Prisla Ucker Calvetti, Rachel Nunes Lorenzoni, Gisele Keller da Rosa, Alexandre Ramos Lazzarotto, Jairo Alberto Dussan-Sarria

**Affiliations:** 1 Post-Graduate Program in Health and Human Development, La Salle University, Canoas, Brazil; 2 Department of Clinical Research Center, Laboratory of Pain & Neuromodulation, Hospital de Clínicas de Porto Alegre, Universidade Federal do Rio Grande do Sul, Porto Alegre, Rio Grande do Sul, Brazil; 3 Post-Graduate Program in Medical Sciences, School of Medicine, Universidade Federal do Rio Grande do Sul (UFRGS), Porto Alegre, Brazil; 4 Pain and Palliative Care Service, Laboratory of Pain & Neuromodulation, Hospital de Clínicas de Porto Alegre, Universidade Federal do Rio Grande do Sul, Porto Alegre, Rio Grande do Sul, Brazil; 5 Surgery Department, Anesthesia and Perioperative Medicine, Hospital de Clínicas de Porto Alegre, Universidade Federal do Rio Grande do Sul, Porto Alegre, Rio Grande do Sul, Brazil; Brain Function Research Group, School of Physiology, Faculty of Health Sciences, University of the Witwatersrand, SOUTH AFRICA

## Abstract

Psychological factors including pain catastrophizing and resilience associate with adjustment and quality of life in people living with chronic pain. Nevertheless, their presentation among females living with HIV and chronic pain has been poorly studied. Given that chronic pain in those living with HIV might occur due to different mechanisms (nociceptive or neuropathic), we hypothesize that the associated psychological states could also differ between these groups. We aimed to compare pain frequency and interference, psychological factors and sleep quality between females living with chronic nociceptive or neuropathic pain. Also, we explored correlations between psychological factors, pain severity and interference in females living with HIV and chronic pain. We performed a cross sectional study assessing females living with HIV and chronic pain, and compared it with a female HIV-positive, pain-free control sample in Brazil. To discriminate the most likely underlying mechanism for the chronic pain, we applied the Leeds Assessment for Neuropathic Signs and Symptoms (LANSS). Forty-nine females living with HIV and chronic pain were assessed, and divided in control (n = 12), nociceptive (n = 10) and neuropathic pain (n = 27) groups. Using validated scales, their pain catastrophizing, resilience, depression, anxiety and sleep disorders were assessed between May 2014 and August 2015. Compared to controls, females living with HIV and neuropathic chronic pain had higher pain frequency (p<0.001), interference on activities (p = 0.002), interference with emotions (p<0.001), catastrophizing (p<0.001), depression (p = 0.015), and lower resilience (p = 0.011). Catastrophizing was also significantly correlated to the burden of chronic pain. The type of chronic pain in females living with HIV should raise concerns regarding significant burden in psychological states in this population (particularly neuropathic pain). Using scales such as the LANSS to identify the type of choric pain, could be of use to address relevant issues for the patients, and to propose tailored therapies.

## Introduction

People living with HIV can experience pain due to different etiologies (*e*.*g*. infections, tumors, myopathies, neurological alterations), and present with a wide spectrum of symptoms that reflect different pain mechanisms: nociceptive, neuropathic or mixed [1]. Pain is experienced by 38% of those suffering sensory neuropathy [2], impairing quality of life [3], daily functioning [4] and employment situation [5]. Such neuropathic lesion have been attributed to both the infection itself and the use of the specific antiretroviral therapy (ART) with dideoxynucleoside analogues [2], because they induce neuronal and axonal mitochondrial DNA damage [6,7]. Although different in their origins, if perseverant both neuropathic and nociceptive pain can unleash peripheral and central nervous system alterations that can translate into chronic pain [8,9], exposing patients to the additional psychological burden that it implies [10].

The prevalence of pain among people living with HIV is estimated to vary between 54% to 83% [11–13], thus it is of great importance for healthcare professionals to better understand the mechanisms involved in chronic pain, as well as the psychological complexities of living with both conditions. In a Brazilian sample, 63.3% of the women living with HIV reported experiencing moderate to severe pain, and was correlated to the stage of infection [14]. Furthermore, being a female living with HIV in this country is an independent risk factor for suffering moderate and severe pain [15]. Nevertheless, the burden of the disease according to the type of pain, has not been explored in South America before.

Chronic pain has been associated with psychiatric disorders (*i*.*e*. insomnia, depression, anxiety, substance dependence and abuse) [2,4,16,17], increasing the burden of the disease, and limiting health improvement more pronouncedly [18]. Chronic nociceptive pain is associated with depression, anxiety, and poor treatment outcomes [19]. Addressing psychological factors is important in the management of chronic pain, including in people living with HIV. Resilience, an ability that promotes adaptive responses to adversities, can independently predict better pain adjustment [20,21]. At the same time, a reduced ability to cope with stressors is related to more depressive symptoms [22]. In individuals living with HIV, resilience is associated with improvement of health [23] because it reduces the negative influence of life’s stressors on physical and emotional symptoms [24]. A community-based prospective cohort study showed that among chronic pain patients, more resilient individuals had a better 10-year survival than non-resilient individuals [25], thus demonstrating the relevance of further assessing this finding. Additionally, besides being prone to objective assessment, resilience is a factor that can be improved, as it has been demonstrated that certain factors such as employment and education help patients become more resilient [26]. Similarly, pain catastrophic is a psychological state that constitutes a risk factor for reduced adherence to medications, and has been correlated with neuropathic pain severity independent of depressive symptoms as well as increased disability due to pain [27].

The psychological factors presented in patients with chronic pain might be related to the underlying pain mechanisms. Nevertheless, these psychological factors of people living with HIV and afflicted by chronic pain have been scarcely studied. Furthermore, the difference in these psychological factors has not been compared between the nociceptive and neuropathic pain chronic pain in HIV before. We hypothesize that people living with HIV and chronic pain might exhibit different levels of resilience and catastrophic thinking according to the presence and type of chronic pain. Thus, we ran a cross-sectional study to explore whether these psychological factors could differ by the most likely etiology of chronic pain (either nociceptive or neuropathic), and compared it to a pain-free sample of people living with HIV, too. We aimed to compare pain frequency and interference, psychological factors and sleep quality between females living with chronic nociceptive or neuropathic pain. Also, we explored correlations between psychological factors, pain severity and interference in females living with HIV and chronic pain.

## Material and methods

### Study design and participants

The methods and results are reported according to STROBE guidelines. All patients provided written informed consent before participating in this observational study, which was approved by the Research Ethics Committee at the University (Institutional Review Board “Comitê de Ética em Pesquisa Unilasalle” IRB 647.372) and was performed in accordance with the Declaration of Helsinki (Resolution 466/12 of the National Health Council). Informed consent was obtained from all individual participants included in the study. In this cross-sectional study, performed between May 2014 and August 2015, we recruited subjects from a convenience sample at a Non-Governmental Organization (NGO) that aided patients with HIV/AIDS in the city of Porto Alegre-RS, Brazil. Before running the current protocol, we performed a small internal pilot to assess feasibility. By that time, we tried to include volunteers irrespective of the sex. Unfortunately, we noticed early that due to different reasons, males living with HIV/AIDS were reluctant to participate. Thus, to favor feasibility we opted to include females only for the study here presented. Researchers responsible for performing the interviews and applying scales approached each subject and invited them to participate, in person, while they were in their leisure time in the NGO. This NGO assists patients with hygiene, group education about HIV, psychological and medical support, relaxing therapies such as massage, and social support with food and educational materials. According to the Brazilian Institute of Geography and Statistics, in 2016 the city of Porto Alegre-RS had 1’481.000 inhabitants. In this region, 1237 new cases of HIV and 1544 of AIDS were notified to the public health authorities between in 2015 [28]. We interviewed females living with HIV/AIDS aging between 18 and 65 years old, who were receiving ART, and asked three screening questions regarding pain: 1) “Are you currently experiencing any pain?”; 2) “Have you felt any pain every day?”; 3) “Has this pain been continuous during the last three months?”. Females who answered affirmatively the three questions were recruited for the “chronic pain” groups, and those who answered negatively to the three of them were recruited as “pain-free” controls. Furthers questions were asked to identify exclusion criteria: active contagious infection (*e*.*g*. fever, active respiratory symptoms, pulmonary tuberculosis, meningitis), history of chronic diseases associated with neuropathic pain such (*i*.*e*. diabetes, systemic erythematous lupus, rheumatoid arthritis, Human T-cell Lymphotropic Virus infection, chronic kidney failure, peripheral vascular insufficiency, meningitis). As mentioned previously, the NGO offered basic medical consultation, so after having participant’s approval, their medical records were consulted to verify the information given by the participants.

### Variables, instruments and assessments

The dependent variables were pain catastrophizing and resilience, and were compared between those with nociceptive and neuropathic pain, and controls. The likely etiology of pain was identified using the Leeds Assessment for Neuropathic Signs and Symptoms (LANSS) [29] in its validated version for the Brazilian Portuguese [30]. It is a relatively simple instrument that can be taken to the bedside, offering good intra-class correlation and fair internal consistency, presenting 85% sensitivity and 80% specificity when compared to experienced clinicians’ judgement [29]. This scale consists of seven items (five symptoms and two signs) with binary answers (yes = 1 or no = 0), which are weighted according to their odds to predict neuropathic pain. Possible scores range from 0 to 24, where scores ≥ 12 indicate that the etiology is likely to be neuropathic in origin, and scores < 12 indicate that a neuropathic etiology is unlikely. The Brazilian Portuguese version of the scale has good intra-class correlation coefficient (r = 0.97), and fair internal consistency (Cronbach’s alpha = 0.67) [30].

Medical records, interviews, questionnaires and tests were applied by researchers and certified nursing assistants who had previously received specialized training to perform the interviews and tests, and to apply properly the questionnaires. Because all the assessment was performed a single visit, researchers were not blinded to the result of the LANSS.

To describe better our sample, we also assessed the characteristics of the pain, sleep quality, depression and anxiety symptoms, using validated scales for the Brazilian population [30–32]. Demographic data and medical comorbidities were assessed using a standardized questionnaire. Patients were asked for the class of ART in use, and this information was verified checking their medical records. CD4 counts were also collected from patients’ medical records.

To assess catastrophic thinking due to chronic pain, we used the Brazilian version of the Pain Catastrophizing scale (PCS), which counts with high validity and reliability (Cronbach’s α = 0.91) [33]. It consists of a 13-items questionnaire, which is completed based on the patients’ thoughts and feelings when they are in pain. Each item is rated on a 5-point Likert-type scale, having the final score (ranges from 0 to 52) by summation of all items, where higher scores denote greater pain catastrophic thinking [34].

The resilience scale (RS) developed by Wagnild and Young in 1993 [35], and validated and adapted to Portuguese by Pesce in 2005 [36], was used. This scale has good internal consistency (Cronbach’s α = 0.85), with construct validity correlated with self-esteem, family supervision, life satisfaction and social support [36]. It consists of a 25-items, each one rated on a 7-point Likert-type scale, offering final scores ranging between 25 to 175 points, where higher scores denote elevated resilience [36].

The characteristics of patients’ chronic pain were assessed using the Profile of Chronic Pain: Screen for a Brazilian Population (B-PCP:S). In brief, this is a 15-item scale that allows for identification of patients’ multidimensional pain experience, assessing its severity (scores ranging from 0–32, Cronbach’s α = 0.76), interference on emotions (range = 0–36, Cronbach’s α = 0.88) and emotional burden (range = 0–25, Cronbach’s α = 0.87). The so called “frequency of pain” domain asks the subject to rate the frequency of any pain, of severe pain, and greatest pain intensity over the past 6 months, providing an idea of the exposure to pain that the subject is suffering [31].

Sleep quality was assessed using the Brazilian version of the Pittsburgh Sleep Quality Index, BR-PSQI, whose internal consistency is considered high (Cronbach’s α = 0.82). It consists of a 19-items questionnaire that assesses seven components. Sleep quality was further categorized as good (score 0–4), bad (score 5–10) or sleep disorder (scores higher than 10) [36].

Depressive symptoms were assessed using the Beck Depression Inventory (BDI-II) [37, 38]. It consists of a self-rating instrument in which 21 statements about depressive symptoms are given to the participants, who is asked to rate in a 0 to 3 ordinal scale considering the last 15 days. Total score ranges from 0 to 63, where scores lower than 13 are considered minimal/no depression; 14–19 mild depression; 20–28 moderate depression; 29–63 severe depression [38, 39].

Anxiety was assessed using the refined version of the State-Trait Anxiety Inventory [32]. Briefly, a state of anxiety (S-Anxiety) refers to the psychological state evoked by acute situation-driven episodes that can fluctuate with time, while anxiety-trait (T-Anxiety) is related to a lifelong pattern (stable personality disposition). The inventory, has demonstrated adequacy (Cronbach alpha = 0.89) [40]. In brief, the S-Anxiety scale provides 13 statements that describe feelings, and subjects are asked to rate how well they describe their thoughts while in pain, according to a 4-point Likert scale: 1) not at all, 2) somewhat, 3) moderately, 4) very much so. During the T-Anxiety scale, participants are asked to rate the frequency of their feelings through 12 different items, using a 4-point Likert scale: 1) almost never, 2) sometimes, 3) often, 4) almost always. For those females living with HIV but without pain, we asked to recall a previous painful experience to answer the B-PCP:S and the PCS.

### Statistical analysis

Conventional descriptive statistics were used to describe the sample. Characteristics of the psychological states of the samples were compared after verifying normality assumptions using the Shapiro-Wilk test. When violating normality, comparisons were performed between groups using the Kruskal Wallis test for continuous variables, and Chi-squared or Fisher’s exact tests for the categorical variables. Post-hoc analyses were adjusted for multiple comparisons using Bonferroni correction. According to the exploratory nature of the study, further pooling of the three groups was performed to study whether the psychological states could associated, and symmetrical Spearman’s correlations were run. A two-sided alpha level (type I error rate) of less than 0.05 was the statistical significance threshold. The data were analyzed using SPSS version 20.0 (SPSS, Chicago, IL).

## Results

**Fig 1:**
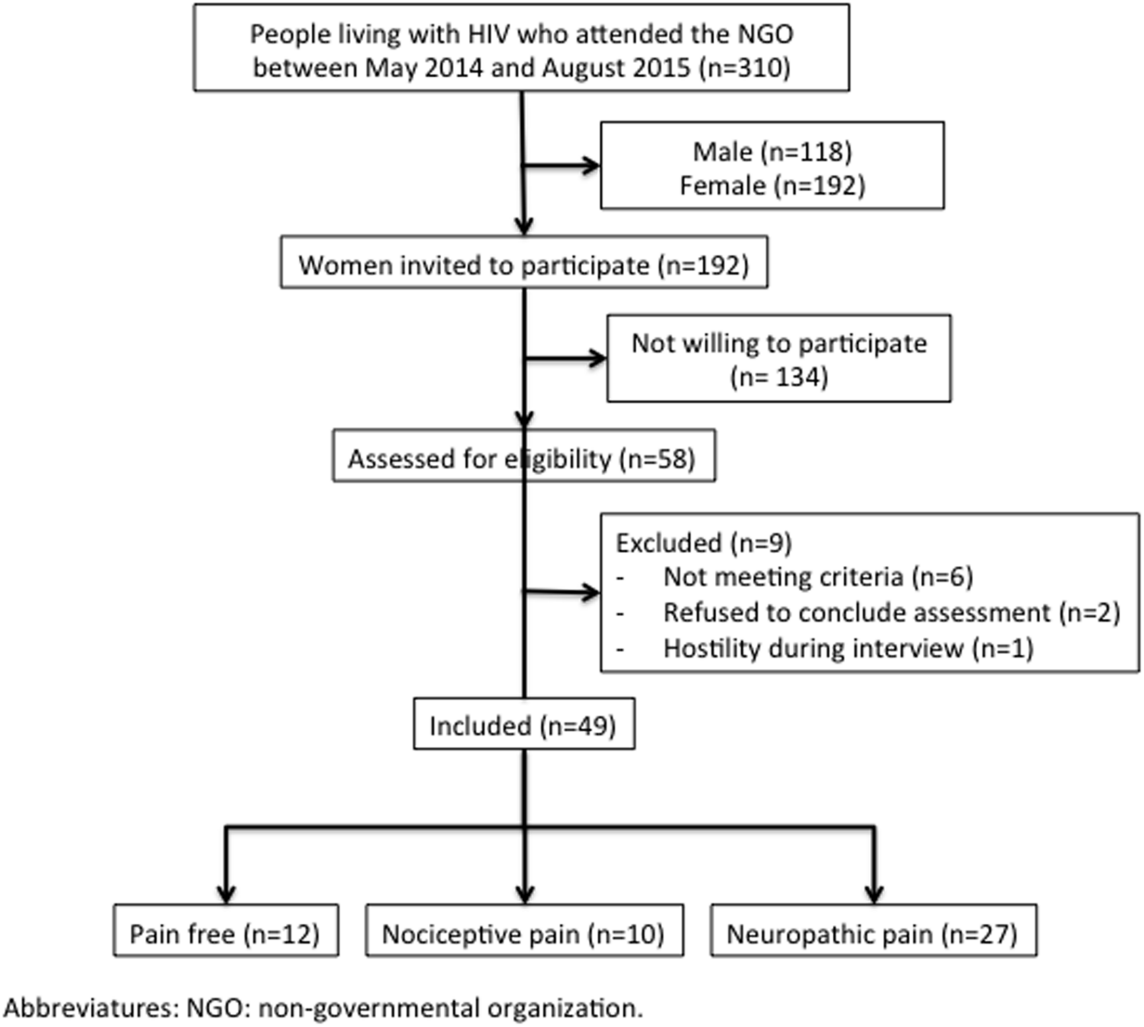
Recruitment flowchart.

Forty-nine patients were included in this study. The recruitment process is summarized in the flowchart presented in Fig 1. The characteristics of the sample are presented in Table 1. To see raw data, see Supporting information (S1 Database). After data collection, according to the LANSS scale, participants were divided into a control group (n = 12) (free of chronic pain); nociceptive pain group (n = 10) (LANSS <12); and a neuropathic pain group (n = 27) (LANSS ≥12). Groups had comparable age, body mass index, education, smoking and alcohol use (P>0.05). Compared to controls, females living with HIV and neuropathic chronic pain presented significantly higher scores in all the domains assessed by the B-PCP:S (frequency of pain, p<0.001; interference on activities, p = 0.002; interference on emotions, p<0.001), higher total catastrophic thinking (p<0.001), including its domains (helplessness, p = 0.016; magnification, p<0.001; rumination, p<0.001) depression symptoms (p = 0.015); and significantly lower resilience (p = 0.011). On the other side, females living with HIV and chronic nociceptive pain, only differed from controls in their anxiety state (p = 0.01). No significant differences were observed between neuropathic and nociceptive pain groups regarding the analyzed psychological states.

**Table 1:** Characteristics of the sample, by presence and type of chronic pain.

Variable	Control (n = 12)	Nociceptive Pain (n = 10)	Neuropathic Pain (n = 27)	P-value
**Age**	46 (38.5;52.5)	42 (39.0;49.0)	46.5 (41.2; 50.7)	0.238^§^
**Body Mass Index**	24.1 (20.4;26.6)	26.9 (20.6;43.5)	26.8 (19.8;30.8)	0.434^§^
**Formal education (years)**	6 (4.5;7.5)	5 (3.0;8.0)	5 (4.7;8.2)	0.980^§^
**Smoking (yes)**	2	0	8	0.388^Ψ^
**Alcohol (yes)**	4	2	9	0.714^Ψ^
**Frequency of Pain (B-PCP:S)**	0 (0.0;9.0)	10 (0.0;26.0)	23.75 (17.7;27.0)*	<0.001^§^
**Interference of Pain on Activities (B-PCP:S)**	0 (0.0;4.5)	0 (0.0;11.0)	16.5 (9.7;23.2)*	0.001^§^
**Interference of Pain on Emotions (B-PCP:S)**	0 (0.0;2.5)	1 (0.0;14.0)	11,5 (6.0;16.2)*	<0.001^§^
**BR-PSQI**				0.020^Ψ^
Good	5	2	3	
Bad	7	5	8	
Sleep Disorder	0	3	16	
**PCS Total**	0 (0.0;15.0)	5 (0.0;15.0)	31.5 (12.5;37.2)*	<0.001^§^
**PCS-Helplessness**	0.0 (0.0;18.0)	14.0 (3.0;18.0)	23.0 (16.0;26.0)*	0.002^§^
**PCS-Magnification**	0.0 (0.0;0.0)	3.0 (0.0;6.2)	9.0 (3.0;11.0)*	<0.001^§^
**PCS-Rumination**	0.0 (0.0;1.0)	4.5 (0.2;8.2)	11.0 (6.0;14.0)*	<0.001^§^
**Resilience Scale**	155.5 (147.5;167.2)	148.0 (126.0;154.0)	138.0 (134.0;149.0)*	0.011^§^
**BDI-II**	5 (4.5;9.5)	12 (3.0;27.0)	17.5 (10.0;26.0)*	0.048^§^
**State anxiety on STAI**	24 (21.5;28.5)	29 (27.9;33.0)*	27 (25.0;29.0)	0.038^§^
**Trait anxiety on STAI**	22 (18.5;23.5)	24 (20.0;26.0)	23.5 (22.0;28.5)	0.363^§^

Values describes as the median (interquartile 25;75) or frequency (n = 49). B-PCP:S: Profile of Chronic Pain: Screen for a Brazilian Population, PCS: pain catastrophizing scale validated for the Brazilian population, BDI-II: Beck Depression Inventory, STAI: State-Trait Anxiety Inventory.

^§^Kruskal-Wallis test,

^Ψ^Fisher’s exact test.

*Significantly different to control group after adjusting for multiple comparisons.

In our sample, females living with HIV had comparable median CD4+ counts (controls, 485 cell/mm^3^; nociceptive pain, 589 cell/mm^3^; neuropathic pain, 496 cell/mm^3^), and median time using ART (controls, 144 months; nociceptive pain, 156 months; neuropathic pain, 120 months). All participants were on ARVs, most typically a NRTI with either a NNRTI or PI.

Finally, in those with pain (either nociceptive or neuropathic) we determined correlations between psychological factors and intensity and interference of pain (Table 2). PCS was significantly correlated to the burden of chronic pain detected by the B-PCP:S in all its domains: moderately associated with the frequency of pain, and strongly related to pain interference on activities and emotions. PCS was moderately associated with depressive symptoms (BDI-II). Also, resilience was weakly correlated to depressive symptoms.

**Table 2:** Correlations between psychological states and pain in females living with HIV (n = 37).

Variable	Frequency of Pain	Interference of Pain on Activities	Interference of Pain on Emotions	BDI-II	Resilience
**PCS total**	0.467**	0.761**	0.718**	0.545**	-0.201
**PCS Helplessness**	0.356*	0.356*	0.560**	0.524**	-0.108
**PCS Magnification**	0.403*	0.584**	0.668**	0.545**	-0.146
**PCS Rumination**	0.364*	0.676**	0.585**	0.508**	-0.240
**State anxiety on STAI**	-0.033	-0.263	-0.164	-0.159	0.177
**Trait Anxiety on STAI**	0.159	0.145	0.296	0.300	0.083
**BDI-II**	0.224	0.474**	0.596**	---	-0.455**
**Resilience**	0.191	-0.277	-0.287	-0.455**	---

The data is presented as Spearman’s Rho.

*P-value<0.05,

**P-value<0.001.

## Discussion

In the present study, for the first time in Brazil, we explored whether psychological factors could differ by the most likely etiology of chronic pain (either nociceptive or neuropathic), and compared it to a pain-free sample of females living with HIV. We compared pain frequency and interference, psychological factors and sleeps quality between females living with chronic nociceptive or neuropathic pain, and explored correlations between psychological factors, pain severity and interference with life. We observed that these psychological factors presented with different intensity according to the probable mechanism underlying the chronic pain. Females living with HIV and chronic neuropathic pain suffered the most, presenting more catastrophizing, higher depression scores, more sleep disturbances, and less resilience. On the other side, females living with HIV and chronic nociceptive pain, only differed to those without pain in their higher anxiety states. The exploratory correlation analysis between psychological factors and pain found that the burden of chronic pain (*i*.*e*. its frequency and interference on emotions and activities) was strongly correlated to catastrophizing.

The burden of pain is in part assessed by the B-PCP:S. We observed greater interference of neuropathic pain on activities and emotions. Such burden is not specific for women living with HIV and chronic pain. Although using different assessment tools, as it has been consistently described by other authors that neuropathic pain is related to higher psychological burden [41]. On the other hand, there is a paucity of scientific literature exploring chronic nociceptive pain in people living with HIV, highlighting the relevance of our data, and limiting our ability to discuss our findings.

In our sample, those with chronic neuropathic pain presented elevated catastrophic thinking. Although not in samples with HIV, other authors have previously described an association between catastrophizing and poor outcomes in subjects with chronic pain, including lower quality of life [42], reduced strength and tolerance to physical activity [43, 44], and greater disability [42]. Considering these associations with unfavorable outcomes, great attention should be pain to patients presenting elevated catastrophic thinking. Unfortunately, to the best of our knowledge, PCS works more like a red flag than as a condition to be treated, as trials to manipulate pain catastrophizing have not been successful [45].

Although depression is the most common neuropsychiatric complication among people living with HIV [15], in our sample we observed that only those with neuropathic chronic pain presented more depression symptoms than controls. Although those with nociceptive chronic pain had elevated scores too, they were not significantly different to controls. Such findings suggest some sort of spectrum of the burden of the disease, and reinforce how neuropathic pain symptoms seem to involve more suffering than nociceptive chronic pain. Interesting, depressive symptoms relation to other psychological factors does not seem to be straightforward. It was reported by Lucey et al. [27], that the effect of catastrophizing neuropathic pain was independent of depressive symptoms. Nevertheless, in our study, we observed a significant association between PCS and depressive symptoms (Table 2). Differences between samples could help explain such divergence, because our sample was restricted to females, while 85% males composed Lucey’s sample. As mentioned earlier, PCS was correlated to depression, which has previously been consistently reported among people living with HIV and chronic pain by Uebelacker et al. [4], thus reaffirming the importance of addressing this entity whenever facing elevated PCS in this population.

Our sample with neuropathic pain also presented significant sleep disorders. Such observation was in line with a report from other authors that also observed an association between sleep disorders and pain, showing that the higher the score on the Pittsburgh Sleep Quality Index, the more severe the pain was reported [46]. Given the cross-sectional nature of our study design, it is not possible to define whether pain caused sleep disorders, or vice-versa. Future studies addressing this issue are still necessary.

We observed that patients with neuropathic pain presented with reduced resilience in comparison to those with nociceptive or no pain at all. In our sample, resilience was not associated with pain, catastrophizing or anxiety, but with depression symptoms. Interestingly, a South African sample presented a similar pattern, where resilience was not associated with pain nor physical activity, but showed a direct correlation with quality of life [47]. Furthermore, it has been shown that more resilient patients also have better pain attitudes and beliefs, reduced catastrophizing, and improved social responses to pain, which is translated into better health care and medication utilization patterns [20,48]. Thus, addressing resilience (as a factor independent of pain and anxiety) should be considered part of a high quality of care for people living with HIV.

Taken together, the scientific literature supports the inclusion of the psychological factors in the assessment and controlled treatment of patients with chronic pain. However, it had not been clearly elucidated how their manifestation might vary according to the underlying mechanisms responsible for the chronic pain complaints. Although both mechanisms (neuropathic and nociceptive) can induce sensitization of the central and peripheral nervous system [8], the way that these processes install and persist might allow patients to modulate their psychological adaptation in distinct ways, potentially molding different psychological profiles.

Few differences were observed in the characteristics related to CD4+ counts and type of ART in use between groups. Thus, clinicians should not rely on the type of ART to presume the existence of chronic pain. Although neuropathic chronic pain is more prevalent, there should always be a directed anamnesis trying to elucidate the most likely mechanisms involved to provide tailored therapies. Patients might have comparable ART, but some of them might not have neuropathy symptoms at all and still be experiencing chronic pain (of nociceptive nature, in this case).

Our exploratory study should be interpreted with care regarding some issues. For the reasons exposed in the materials and methods section, our sample was composed of women only. Nevertheless, it is important to remember that there are some differences in experimental pain between males and females, in the sense that there is greater pain sensitivity among females, for reasons not clearly defined yet. Nonetheless, the evidence regarding differences in endogenous pain modulation, functional brain imaging, and response to pain treatments remain inconsistent [49]. Thus, the reader should be discouraged of extrapolating our findings for a male population.

As presented earlier in this manuscript, our exploratory study was run in Porto Alegre, a city of about 1.5 million inhabitants. Thus, the representativeness of our sample should be interpreted considering the size and characteristics of its HIV/AIDS population. In our sample, the median time of use of ART was 10 to 13 years, making it reasonable to think that they were diagnosed by that time. Analyzing official data regarding HIV/AIDS in Porto Alegre [28], there were 10,955 new cases and 3,157 deaths (only those having HIV/AIDS among main diagnoses) between 2007 and 2011. Considering a male/female ratio of 1.3 it is estimated that 3,390 females diagnosed in that period could still be alive in Porto Alegre by the time we run our study. This would mean that we probably assessed around 5.6% of the population of females living with HIV/AIDS who started ART between 2007 and 2010 in Porto Alegre. About 2.4% of our sample was free of chronic pain, which should raise concern as it suggests that most of the females living with HIV/AIDS are also suffering some type of pain. Even so, the size of our sample is still relatively small for making generalizations. Nevertheless, we recruited a sample who fit through the rigorous criteria to warrant internal validity so we could allege with greater certainty which mechanism (either nociceptive or neuropathic) was more likely involved, and which psychological characteristics accompanied each one of them. It was of great relevance to exclude subjects with comorbidities that could act as confounders due to its known association with neuropathic pain, such as those with diabetes, lupus, HTLV, chronic kidney disease. Further studies recruiting greater samples (possibly collaborative studies) are necessary to validate our findings.

Finally, these findings highlight the importance for close clinical evaluation of patients with chronic pain and HIV. Besides a detailed clinical evaluation, the use of validated scales (e.g. LANSS) are very important to determine the type of pain and can be crucial when defining the etiology of chronic pain, which should raise alerts regarding potential alterations in psychological states. We observed the relationship between psychological states and pain. Females living with HIV and with a neuropathic pain etiology presented with higher burden in the studied psychological states, while those with a nociceptive pain component presented with a less ill profile. Correctly characterizing the type of pain should motivate the caregivers to offer tailored therapies.

## Supporting information

S1 Database (SAV)
